# The Poor Man's Home.—XVIII

**Published:** 1897-12-18

**Authors:** 


### Modern Sociology.

### THE POOR MAN'S HOME.—XVIII.

### Housing in America (*concluded*).

The Tenement House Building Company of New York supplies a cheaper kind of dwelling than Mr. White. Its dividends are limited to 4 per cent. Profits beyond this figure are set aside to form a reserve fund, and also to permit of rebates being given tenants in cases of sickness or loss of work, in which circumstances great leniency is shown. The buildings of the company are on the east side, in one of the lowest and most densely-peopled districts in New York. From the fact that the majority of the tenants are Russian Jews, it will be inferred that they are mostly of the very poor, and also that their habits in the matter of cleanliness leave a good deal to be desired. They are, therefore, of the class whose welfare cannot be entirely left to the operation of ordinary commercial laws.

The buildings are certainly less luxurious than the Riverside Buildings. A much larger proportion of the ground is built on—70 per cent., as against 50 per cent. The remaining space is given up to the tenants for a drying-ground and playground for the children. This space is paved with granolithic, and is swept and washed twice a day. Granolithic is used also for paving the cellars, which are lighted from a space 4 ft. wide, which has been left between the building line and the house itself. This area is protected by an iron grating. Part of the cellars are used as laundries and bath-room, and the remainder is partitioned off to form storage places for fuel, &c. The buildings are six storeys high, and are solidly built. They are as far as possible fire-proof, and fire-escapes are placed at the back of the houses; besides which, iron bridges connect each of the three blocks into which the building is divided. There are in all 104 lodgings in the building—43 of two rooms and 61 of three. Some of the latter are, however, so arranged that they can be divided into a tenement of two and another of one room. The company provides gas in the passages and steam heating in the winter. One of the rules of the company is that no tailoring or other shop work is to be done in the houses, and also that the tenants are not to keep lodgers. This latter provision is, however, systematically evaded, and if any objection is made the lodger is declared to be a relative. Elevators exist for the purpose of taking up and down heavy loads, such as fuel, washing, food, &c. Ashes and garbage are taken down to cans under the pavement, and from them are emptied into the garbage carts by the city scavengers. Each apartment has a clothes press, and there is a cupboard in every kitchen, placed over the sink. The ceilings are 10 ft. high on the first floor, and 9 ft. throughout the rest of the building. In a two-room flat, the kitchen, as a rule, measures 14 ft. by 10 ft., and the bedroom 10 ft. by 9 ft. The three-room lodgings are of various sizes. The largest has the living room 16 ft. by 10 ft., one bedroom 12 ft. by 8 ft., and the other 13 ft. by 9 ft. In others the living-room measures 14 ft. by 10 ft., and the bedrooms each 10 ft. by 8 ft. The sanitary arrangements are good enough, but not of the best. Water-closets are provided in the proportion of one for every two families. They are placed in the staircases, or opposite the landings. They do not receive light or ventilation from the open air. One only in a group is so favoured, and the light it receives is communicated by transoms to the others. A ventilating shaft is placed outside this one window, and flues convey air to the several closets. The closets themselves are, however, of good construction, and are well flushed. The plumbing throughout is good, and an unlimited quantity of water is allowed. The health record is certainly excellent. There have been no epidemics, and in five years only six adults and five children have died.

The rent of a two-room flat is 8 dols. a month on the first and second floors, 7½ dols. on the third and fourth, and 7 dols. on the fifth and sixth (£1 13s. 4d., £1 11s. 3d., and £1 9s. 2d.) There are also some smaller tenements at 6½ dols. (£1 5s.) The three-room flats vary considerably in rent, according to the advantages of size and situation. At the front of the building they run from 14 dols. in the first storey to 12 dols. in the two highest per month (£2 18s. 4d. to £2 10s.) Rents are supposed to be received monthly in advance, but, as a matter of fact, instalments are accepted at any time during the current month, and considerable leniency is shown in the matter of arrears. An average loss of 5 per cent. of the rental is usual through this cause. One part of the building is rented by a philanthropic society as a kindergarten, where instruction is given free, but a charge of two cents (1d.) a day is made for lunch. There are six baths in the basement for the use of tenants, which are free, and are fairly well patronised.

The Rufus Ellis Memorial Building, in Boston, is a tenement much like the one we have been describing, but the rents are a little lower than in New York. It also pays about 4 per cent. to its shareholders, and puts aside from 1¼ to 1½ per cent. for reserve. The building is four storeys high. It contains a fair number of one-room tenements. These measure, as a rule, 14 ft. by 11 ft. The average rent of these is a dollar (4s. 2d.) a week. Two-room flats contain a living-room 13 ft. long by 10 ft. wide, and bedrooms 14 ft. long by 7 ft. or 8 ft. These cost 1½ dols. to 1 dol. 80c. weekly (6s. 3d. to 7s. 6d.) There are some cheap three-room flats, costing only a dollar a week, but these are not very desirably situated; the majority rent at 2½ dols. (10s. 6d.) The rooms are rather smaller, and the ceilings lower, than those we have been describing in New York. In other respects they seem to be very similar; the sanitary arrangements have the same advantages and the same defects. Rents are paid weekly in advance; three weeks' arrears are generally allowed before eviction, and, we are told, "tenants almost invariably do not pay the last week's rent before moving out." The rents of some of the lodgings have been reduced for one reason or another, but they are still about 3½d. a room higher than those of wooden houses in the neighbourhood. The latter are, however, inferior to the Rufus Ellis dwellings in every respect.

In some of the smaller towns in the States employers have built cottages for their workers. These are, as a rule, constructed of wood, and very fair accommodation is provided at a moderate cost. But there is no general impulse on the part of employers to look upon the housing of employés as part of their duty. The experience of many firms who have provided libraries, recreation-rooms, &c., has been depressing; and the general impression seems to be that the American would rather be uncomfortable in his own way than comfortable in his master's.

